# Utility of the Mini-Cog for Detection of Cognitive Impairment in Primary Care: Data from Two Spanish Studies

**DOI:** 10.1155/2013/285462

**Published:** 2013-08-28

**Authors:** Cristóbal Carnero-Pardo, Isabel Cruz-Orduña, Beatriz Espejo-Martínez, Carolina Martos-Aparicio, Samuel López-Alcalde, Javier Olazarán

**Affiliations:** ^1^Neurology Service, Virgen de las Nieves University Hospital, Granada, Spain; ^2^FIDYAN Neurocenter, Granada, Spain; ^3^Neurology Service, Infanta Leonor Hospital, Madrid, Spain; ^4^Neurology Service, La Mancha Centro Hospital, Alcázar de San Juan, Spain; ^5^Hermanos Sangro Specialties Clinic, Neurology Service, Gregorio Marañón General University Hospital, Madrid, Spain; ^6^Alzheimer Disease Research Unit, Alzheimer Center Reina Sofia Foundation-CIEN Foundation, Carlos III Institute of Health, Madrid, Spain

## Abstract

*Objectives*. To study the utility of the Mini-Cog test for detection of patients with cognitive impairment (CI) in primary care (PC). *Methods*. We pooled data from two phase III studies conducted in Spain. Patients with complaints or suspicion of CI were consecutively recruited by PC physicians. The cognitive diagnosis was performed by an expert neurologist, after formal neuropsychological evaluation. The Mini-Cog score was calculated *post hoc*, and its diagnostic utility was evaluated and compared with the utility of the Mini-Mental State (MMS), the Clock Drawing Test (CDT), and the sum of the MMS and the CDT (MMS + CDT) using the area under the receiver operating characteristic curve (AUC). The best cut points were obtained on the basis of diagnostic accuracy (DA) and kappa index. *Results*. A total sample of 307 subjects (176 CI) was analyzed. The Mini-Cog displayed an AUC (±SE) of 0.78 ± 0.02, which was significantly inferior to the AUC of the CDT (0.84 ± 0.02), the MMS (0.84 ± 0.02), and the MMS + CDT (0.86 ± 0.02). The best cut point of the Mini-Cog was 1/2 (sensitivity 0.60, specificity 0.90, DA 0.73, and kappa index 0.48 ± 0.05). *Conclusions*. The utility of the Mini-Cog for detection of CI in PC was very modest, clearly inferior to the MMS or the CDT. These results do not permit recommendation of the Mini-Cog in PC.

## 1. Introduction

The aging of the population has come along with an increase in the incidence of cognitive impairment (CI) [1], a clinical syndrome that, in about one-third of the patients, precedes dementia [2]. An early detection of CI could produce benefits at different levels, including early dementia diagnosis, access to treatments, and delay or even reversion of cognitive deterioration [3–5].

 Primary care (PC) presents optimal characteristics of accessibility and continuity of care, which are essential for early detection and management of CI [6]. In this vein, the focus of the PC physicians should be the detection of CI, rather than dementia. A separation of mild cognitive impairment (MCI) and dementia would not only be difficult or arbitrary in many instances but would also lead to missing opportunities for treatment and research [7].

 The detection of CI requires a proactive attitude and the use of cognitive tests. In PC, cognitive tests need to be brief and easy to administer and interpret. In addition, these tests should have been specifically validated in the PC setting, with an adequate control of the potential influence of age, education, and other social variables [8]. Albeit not simple, rather long, and very influenced by education, the Mini-Mental State (MMS) [9] is still the most used cognitive test. As additional limitations, the MMS displayed modest diagnostic utility for detection of CI in PC [10, 11] and was recently protected by copyright [12].

The Mini-Cog is a very simple and brief cognitive test that comprises a three-item verbal memory task and a simplified evaluation of the Clock Drawing Test (CDT). According to their authors, the Mini-Cog is not influenced by education [13]. Indeed, in a population-based study, this test was as effective as a formal neuropsychological battery for detecting dementia [14]. The Mini-Cog was also well accepted by the patients [15], displayed good correlation with the MMS [16], and was recently recommended in some consensus guidelines and reviews [17–20]. However, there are very few studies addressing the yield of the Mini-Cog for detection of CI (i.e., both MCI and dementia), which displayed conflicting results [21, 22].

The objective of the present study was to evaluate the diagnostic utility of the Mini-Cog for detection of CI in patients who presented with complaints or suspicion of cognitive deterioration in PC. 

## 2. Methods 

 We analyzed data from two phase III studies of evaluation of diagnostic instruments [23] that were conducted in two different cities of Spain (i.e., Madrid and Granada). The study of Madrid was conducted in the Hermanos Sangro health center (Health District 1, Autonomous Community of Madrid, urban area), and the study of Granada was conducted in the Almanjáyar/Cartuja, the Casería de Montijo, and the Salvador Caballero health centers (Health District Granada Norte, Autonomous Community of Andalucía, urban area). The methods of these studies were described in detail elsewhere [24, 25]. In the two studies, patients and caregivers were informed of the procedures and purpose of the studies and gave their consent to participate. Other essential common characteristics of the two studies were as follows.

### 2.1. Setting

The recruitment of patients and the administration of the screening instruments were performed in PC, whereas the formal neuropsychological evaluation and the final diagnosis (gold standard) were performed in specialized care (SC), which replicates the standards of clinical practice.

### 2.2. Recruitment

The patients were prospectively and consecutively recruited by the PC physicians. All the subjects who presented with complaints or suspicious (either by informant or by family physician) regarding cognitive dysfunction or cognitive deterioration were invited to participate in the study. Patients with a former diagnosis of CI were not included. 

### 2.3. Screening Instruments

A validated Spanish version of the MMS was utilized, with the modification of ignoring the possibility of spelling world backwards [26]. The CDT was scored from 0 to 7 [27]; in one of the sites (Granada), the requested time was “ten past eleven,” whereas in the other site (Madrid) the requested time was “twenty to four.” Otherwise, the instructions for the clock drawing test in the two sites were alike (i.e., “please, draw a big round clock, put all the numbers on it, and display the hands on (requested time)”). The score of the Mini-Cog was obtained *post-hoc*, for the purpose of the present investigation. One point was given for every recalled word from the MMS delayed recall task, and two additional points were given if the clock fulfilled the following conditions: (a) all the numbers from 1 to 12 were included and there was no repeated number, (b) all the numbers were in the right order and direction, and (c) the hands unequivocally displayed the requested time. Hence, the score of the Mini-Cog varied from 0 to 5 [21].

### 2.4. Cognitive Diagnosis (Gold Standard)

Mild cognitive impairment (MCI) was diagnosed on the basis of the following criteria: (a) an abnormal performance was documented in at least one neuropsychological test; (b) that abnormal performance was deemed to be of clinical relevance (i.e., not due to low premorbid level, lack of motivation, etc.), and (c) dementia criteria were not fulfilled. The diagnosis of dementia was made according to the 4th edition, text revised, of the Diagnostic and Statistical Manual of Mental Disorders (DSM-IV-TR) [28]. The cognitive diagnosis was performed by two senior neurologists with expertise in cognitive and behavioural neurology (CC, JO) after medical visit, neurological exam, mental status exam, and formal neuropsychological evaluation, which included tests of memory, attention/executive functions, language, and visual-spatial abilities [24, 25]. Throughout the study, laboratory determinations and other ancillary tests (e.g., cranial computed tomography scan) were performed as the clinical situation indicated.

### 2.5. Independence of the Cognitive Diagnosis

The results of the screening instruments were not utilized for the cognitive diagnosis in the original studies. However, for the present investigation, the CDT, which was a screening instrument in the study of Granada but was included in the formal neuropsychological evaluation in the study of Madrid, was analyzed as a screening instrument. 

### 2.6. Double Blinding

The screening instruments were administered before the neuropsychological and neurological exams were conducted, in different settings (PC versus SC) and by different professionals. The neurologists, who performed the cognitive diagnosis, were blind to the results of the screening instruments with the exception of the CDT in the study of Madrid, as mentioned in the previous paragraph.

### 2.7. Reference Bias

Complete verification was conducted; that is, all the recruited patients received the same neuropsychological and neurological assessment and all of them received a final cognitive diagnosis, independently of the results in the screening instruments [29].

 There were some differences between the two studies regarding age and period of inclusion. In the study of Madrid, only ≥50 year old patients were recruited, and the period of inclusion ran from April 1, 2000 to October 31, 2002 (i.e., 31 months). There was no age limit in the study of Granada and the period of inclusion of that study ran from February 1, 2008 to January 31, 2009 (i.e., 1 year). The study of Granada was approved by the Hospital Virgen de las Nieves Ethics Committee, and participants signed informed consent. In the study of Madrid, only verbal consent was required from patients and caregivers/informants.

*Statistical Analysis*. Bivariate comparisons between quantitative variables were performed using Student's *t*-test, whereas qualitative variables were compared using chi-square and Fisher exact tests. A potential influence of site was specifically addressed by means of two-factor (site [Granada versus Madrid] × cognitive diagnosis [CI versus no CI]) analysis of covariance (ANCOVA) adjusted for age (years), sex (man versus woman), and education (no or incomplete primary education versus primary or higher education). 

 The diagnostic utility of the Mini-Cog, the CDT, the MMS, and the sum of the MMS and the CDT scores (MMS + CDT) was evaluated and compared for detection of CI (MCI or dementia) using receiver operating characteristic (ROC) curves. Differences between the areas under the curve (AUC) were analyzed using the Hanley and McNeil correction to compare diagnostic tests in the same sample [30]. In addition, the sensitivity (Sn), the specificity (Sp), the positive likelihood ratio (LR+), the diagnostic accuracy (DA) (proportion of patients correctly classified), and the *kappa* index of diagnostic concordance were obtained for all the cut points in all the tests. The best cut point was chosen for each test on the basis of maximization of DA and *kappa* index. 

 The interrater reliability of the Mini-Cog was evaluated in a subsample of 50 subjects (first 25 subjects included in each site). Four evaluators that were blind to one another and to all other study variables scored the clocks and the three-item recall tasks of those subjects, and the intra-class correlation coefficient (ICC) was calculated. 

 All the statistical tests were two tailed, and a level of *P* < 0.05 was chosen for statistical significance. Statistical analyses were performed using the SPSS 15.01 and MedCalc 12.3 software.

## 3. Results

 The demographic and clinical characteristics of the total sample and of the two original samples are presented in Table 1. Overall, there was predominance of female sex (72.0%), and the educational achievement was low (50.8% of the participants had not complete primary education). The frequency of illiteracy was particularly high in the participants from Granada (14.1% versus 4.8%, *P* ≤ 0.009). Patients from Granada also presented with a higher frequency of dementia (34.5% versus 9.1, *P* ≤ 0.001) and obtained lower scores in all the cognitive tests (Table 1).

**Figure 1:**
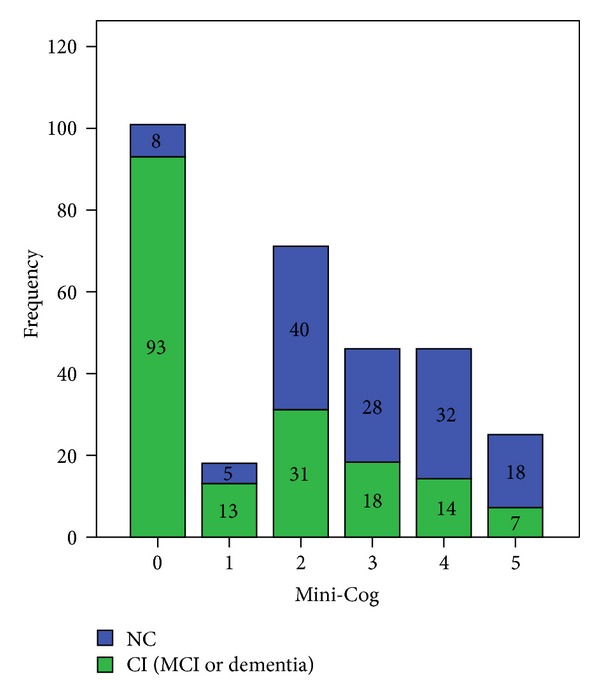
Frequency of Mini-Cog individual scores according to cognitive diagnosis. CI: cognitive impairment; MCI: mild cognitive impairment; NC: normal cognition.

 As it was expected, the subjects with MCI or dementia were older than the subjects with normal cognition (NC). The subjects with CI had also less educational achievement and scored lower in all the cognitive tests (Table 2). There were no sex differences across the different cognitive diagnoses.  Figure 1 represents the distribution of the Mini-Cog score in the two study groups.

 The score of the Mini-Cog was significantly influenced by age (*P* ≤ 0.001) and education (*P* ≤ 0.002) but not by sex (*P* = 0.99) or study site (*P* = 0.18). A total of 16 participants belonging to the sample of Granada (11.3% of patients from Granada, 5.2% of the total sample, and 11 of them illiterates) did not even try to draw the clock, in which case a zero score was given. 

**Figure 2:**
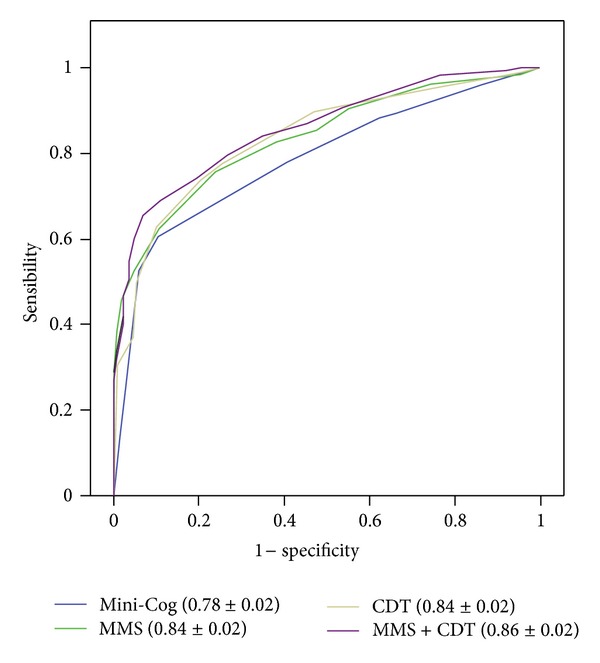
ROC curves for detection of CI (MCI or dementia). The area (±SD) under the receiver operating characteristic curve (ROC) is indicated. MMS: Mini-Mental State; CDT: Clock Drawing Test; CI: cognitive impairment; MCI: mild cognitive impairment.

 The diagnostic yield of the Mini-Cog was inferior (AUC ± SE 0.78 ± 0.02), compared to that of the MMS (0.84 ± 0.02, *P* ≤ 0.001), the CDT (0.84 ± 0.02, *P* ≤ 0.001), and the MMS + CDT (0.86 ± 0.02, *P* ≤ 0.001) (Figure 2). The best cut point of the Mini-Cog was 1/2, with Sn 0.60 (confidence interval (CI) 95% 0.53–0.67), Sp 0.90 (CI 95% 0.84–0.95), LR+ 6.1 (CI 95% 3.6–0.3), DA 0.73, and kappa index 0.48 ± 0.05). The usually recommended cut point of the Mini-Cog of 2/3 displayed an inferior utility, with Sn 0.78 (CI 95% 0.71–0.84), Sp 0.59 (CI 95% 0.51–0.68), DA 0.70, and kappa index 0.38 ± 0.04 (Table 3). Using a cut point of 22/23, the MMS had similar Sn (0.76, CI 95% 0.68–0.82), but better Sp (0.76, CI 95% 0.68–0.83) and DA (0.76). As for the CDT, a cut point of 5/6 achieved similar results to those of the MMS, with Sn 0.76 (CI 95% 0.68–0.82), Sp 0.78 (CI 95% 0.70–0.85), and DA of 0.76. The MMS + CDT was slightly, but not significantly, superior to the MMS or the CDT alone (Table 4, Figure 2). 

 The Mini-Cog displayed an excellent interrater reliability, with an ICC (±SD) of 0.97 ± 0.01.

## 4. Discussion

 We pooled data of two homogeneous studies to ascertain the value of the Mini-Cog for detection of CI (either MCI or dementia) in PC. The score of the Mini-Cog was obtained *post-hoc*, utilizing data from the MMS and the CDT. The diagnostic utility obtained by the Mini-Cog was low, with an AUC (±SE) of 0.78 ± 0.02 and a percentage of correctly classified subjects of 70%, when the recommended cutoff of 2/3 was applied [21]. A cut point of 1/2 obtained a slightly better yield (73% of cases correctly classified) on the basis of a good specificity (0.90 (CI 95% 0.84–0.95)) but a low sensitivity (0.60 (0.53–0.67)), which was unacceptable for a detection test.

The diagnostic utility of the Mini-Cog was inferior to that of the MMS, which was moderate (AUC 0.84 ± 0.02). When the best MMS cut point was applied (i.e., 22/23), the sensitivity and specificity were modest (0.76 (0.68–0.82)), and the percentage of cases correctly classified did not reach 80% (DA 0.76) (Table 4). These modest results of the MMS coincide with other study conducted in PC [10] and with the results from two systematic reviews [11, 18]. Albeit also modest, the results obtained by the CDT in the present investigation were superior to the results obtained in previous studies [31] that led to disregard of the CDT for detection of MCI [32]. A possible explanation for our better results with the CDT is that this test was included, in one of the study sites, in the reference diagnosis. We also evaluated the diagnostic utility of the sum of scores of the MMS and the CDT, but there was no significant gain, with a clear increase in testing time, when compared to the MMS or the CDT alone. That result is also in agreement with a previous investigation [33]. 

In previous research, the Mini-Cog displayed good performance for detection of dementia, with figures that were similar [14] or even superior [34] to those obtained by the MMS or the CDT. To the authors' knowledge, only two studies had so far addressed the utility of the Mini-Cog for detection of CI, with conflicting results. In a sample of predominantly male old people, the results of the Mini-Cog were not satisfactory (AUC 0.64) [22], whereas, in a study conducted in a sample of predominantly “unserved ethnic minorities,” a diagnostic accuracy of 0.83 was reported [21]. The proportion of patients with dementia, that differed dramatically in those studies (3.3% dementia, 39.2% MCI in Holsinger's study [22] versus 41.5% dementia, 21.3% MCI in Borson's study [21]), could explain the reported differences in the diagnostic yield of the Mini-Cog. Our results, in a sample of intermediate characteristics (20.8% dementia, 36.5% MCI) (Table 1), were also intermediate in terms of Mini-Cog diagnostic utility (diagnostic accuracy 0.73, AUC 0.78) (Table 3 and Figure 2). Clearly, all these data advise against the use of the Mini-Cog for detection of CI in settings where the suspected proportion of unrecognized dementia is low. 

We found a significant influence of education in the Mini-Cog performance, which was not in agreement with the original validation study [13]. This discrepancy can be explained in the light of the low educational achievement of our sample (Table 1). In the original validation study, all the participants had completed at least six years of education, whereas in the present study, a proportion of 50.8% of the subjects had not complete primary school, which in Spain usually means less than eight years of formal education, and there was 9.1% of illiteracy (Table 1). There is strong evidence of the influence of education and literacy on CDT performance [31, 35], and reasonably this should be the same with the Mini-Cog. In fact, poor yield of the Mini-Cog was previously reported for dementia detection in a sample of low educational level [36]. Overall, these data suggest a negative influence of education on the Mini-Cog, restricted to the most inferior educational strata. 

Our study had some limitations. First, the CDT was part of the reference diagnosis in one of the study sites, a limitation that could have overvalued the utility of the Mini-Cog and therefore would not essentially alter the study conclusions. Second, the score of the Mini-Cog was obtained *post-hoc* from two different tests. This methodology was used by several researchers with exploratory purposes, including the original descriptions of the Mini-Cog [13, 14] to ascertain the validity of the test [34]. Certainly, the obtained results discourage future prospective studies of the Mini-Cog. Third, the present results were derived from two slightly different studies. However, the results obtained by the Mini-Cog in each of the individual studies were similar (data not shown), which strengthens the consistence and the external validity of the presented global results. As additional strength, the two pooled studies fulfilled the standards of high-quality phase III diagnostic studies [37], a consecutive and systematic recruitment was conducted, and there were virtually no exclusion criteria.

## 5. Conclusions

Simplicity and shortness in time are indeed characteristics of the Mini-Cog, which are necessary for use in PC. However, the Mini-Cog was not a valid choice in the studied sample, and this result, which was consistent with previous research, advise against the use of the Mini-Cog, particularly in settings where the proportion of unrecognized dementia is low. In these settings, the use of tests that offer a wider range of tasks and score, yet maintaining acceptability and feasibility, seems more adequate [38]. In light of the relevance of the detection of CI, more human and technical resources should be implemented in PC. 

## Figures and Tables

**Table 1:** Demographic and clinical characteristics of the total study sample and of the two original samples.

	Total	Madrid	Granada	*P* value
*n*	307	165	142	
Female gender	221 (72.0)	118 (71.5)	103 (72.5)	0.90
Age, years	72.0 ± 10.1	71.9 ± 8.9	72.1 ± 11.4	0.84
Educational level				
<Primary	156 (50.8)	84 (50.9)	72 (50.7)	1.00
≥Primary	151 (49.2)	81 (49.1)	70 (49.3)	
Cognitive diagnosis				
NC	131 (42.7)	75 (45.5)	56 (39.4)	≤0.001
MCI	112 (36.5)	75 (45.5)	37 (26.1)	
DEM	64 (20.8)	15 (9.1)	49 (34.5)	
Mini-Cog	2.0 ± 1.7	2.4 ± 1.7	1.5 ± 1.5	≤0.001
MMS	21.3 ± 5.2	22.3 ± 4.5	19.9 ± 5.7	≤0.001
CDT	4.5 ± 2.5	5.0 ± 2.0	4.0 ± 2.8	≤0.001
MMS + CDT	25.7 ± 7.2	27.3 ± 6.0	23.9 ± 8.1	≤0.001

Data represent number of individuals (%) or mean ± SD. MMS: Mini-Mental State; CDT: Clock Drawing Test; DEM: dementia; NC: normal cognition; MCI: mild cognitive impairment.

**Table 2:** Demographic characteristics and test results by cognitive diagnosis.

	NC	CI	MCI	DEM	*P* value*
*n*	131	176	112	64	
Female gender	100 (76.3)	121 (68.8)	74 (66.1)	47 (73.4)	0.09
Age, years	66.9 ± 10.4	75.8 ± 8.1	73.6 ± 8.1	79.6 ± 6.5	≤0.001
Educational level					
<Primary	48 (36.6)	108 (61.4)	63 (56.3)	45 (70.3)	≤0.001
≥Primary	83 (63.4)	68 (38.6)	49 (43.8)	19 (29.7)	
Mini-Cog	2.9 ± 1.3	1.2 ± 1.5	1.7 ± 1.7	0.4 ± 0.8	≤0.001
MMS	24.6 ± 3.0	18.7 ± 5.1	21.2 ± 4.0	14.2 ± 3.8	≤0.001
CDT	6.1 ± 1.3	3.3 ± 2.4	4.4 ± 2.0	1.4 ± 1.9	≤0.001
MMS + CDT	30.7 ± 3.7	22.0 ± 7.0	25.6 ± 5.2	15.8 ± 5.1	≤0.001

Data represent number of individuals (%) or mean ± SD. MMS: Mini-Mental State; CDT: Clock Drawing Test; NC: normal cognition; CI: cognitive impairment (MCI or dementia); DEM: dementia; MCI: mild cognitive impairment.

*For the comparison between NC versus CI (MCI or dementia).

**Table 3:** Utility of the Mini-Cog for the detection of CI (MCI or dementia).

Cutoff	Sn	Sp	LR+	DA	*κ*
≤0	0.53 (0.45–0.60)	0.94 (0.88–0.97)	8.6 (4.4–17.2)	0.70	0.43 ± 0.04
≤1	0.60 (0.53–0.67)	0.90 (0.84–0.95)	6.1 (3.6–10.3)	0.73	0.48 ± 0.05
≤2	0.78 (0.71–0.84)	0.59 (0.51–0.68)	1.9 (1.5–2.4)	0.70	0.38 ± 0.05
≤3	0.88 (0.82–0.92)	0.38 (0.30–0.47)	1.4 (1.2–1.6)	0.67	0.28 ± 0.05
≤4	0.96 (0.92–0.98)	0.14 (0.08–0.21)	1.1 (1.0–1.2)	0.61	0.11 ± 0.04
≤5	1.00 (0.98–1.00)	0.00 (0.00–0.03)	1.0 (1.0–1.0)	0.67	0.00 ± 0.00

DA: diagnostic accuracy (proportion of correct diagnoses); *κ*: kappa index; LR+: positive likelihood ratio; Sn: sensibility; Sp: specificity.

**Table 4:** Utility of the screening tests for the detection of CI (MCI or dementia) using the best cut points.

Test	Cutoff	Sn	Sp	DA	*κ*
Mini-Cog	1/2	0.60 (0.53–0.67)	0.90 (0.84–0.95)	0.73	0.48 ± 0.05
MMS	22/23	0.76 (0.68–0.82)	0.76 (0.68–0.83)	0.76	0.51 ± 0.05
CDT	5/6	0.76 (0.68–0.82)	0.78 (0.70–0.85)	0.76	0.53 ± 0.05
MMS + CDT	25/26	0.65 (0.58–0.72)	0.93 (0.87–0.97)	0.77	0.56 ± 0.05

aROC: area under curve ROC; Sn: sensibility; Sp: specificity; DA: diagnostic accuracy (proportion of correct diagnoses); *κ*: kappa index; MMS: Mini-Mental State; CDT: Clock Drawing Test.
